# Sweet syndrome with osseous and splenic involvement: A case report

**DOI:** 10.1016/j.radcr.2021.10.026

**Published:** 2021-11-12

**Authors:** Cheryl Zhang, Alaa Elmaoued, Benjamin Rincy, Brett Ploussard, Mario Saab-Chalhoub, Anup Jacob Alexander, Emad Allam

**Affiliations:** Department of Radiology and Medical Imaging, Department of Pathology and Laboratory Medicine, Loyola University Medical Center, 2160 S 1st Ave., Maywood, IL 60153, USA

**Keywords:** Sweet syndrome, Acute febrile neutrophilic dermatosis, Crohn's disease, Osteomyelitis, Abscess, SS, Sweet syndrome, GI, gastrointestinal, WBC, white blood cell, CRP, c-reactive protein, ESR, erythrocyte sedimentation rate, AFB, acid fast bacilli, p-ANCA, perinuclear-antineutrophil cytoplasmic antibodies, ANA, antinuclear antibodies, RF, rheumatoid factor, PICC, peripherally inserted central catheter, EKG, electrocardiogram, H&E, hematoxylin and eosin, CRMO, chronic recurrent multifocal osteomyelitis

## Abstract

Sweet syndrome is an uncommon inflammatory skin condition. Here we present a case of Sweet syndrome in a young woman with rare extracutaneous manifestations, including bone and splenic fluid collections, with marked improvement following treatment with systemic corticosteroids. The patient was subsequently diagnosed with Crohn's disease which can be seen in the setting of Sweet syndrome. Sterile abscesses should be considered in patients with a clinical diagnosis of Sweet syndrome and focal symptomatology.

## Background

Sweet syndrome (SS), also known as acute febrile neutrophilic dermatosis, is an inflammatory and/or hypersensitivity reaction that commonly affects the skin and is characterized by a constellation of clinical and histologic findings including fever, neutrophilia, raised erythematous lesions (plaques, nodules or papules), and infiltrates of mature neutrophils in the upper dermis [1]. SS is also reported to have extracutaneous manifestations in the central nervous system, ears, eyes, oral mucosa, various visceral organs, and joints [2,3]. It is rare, however, to find bone involvement in patients without prior malignancy or preceding hematological treatment [14,16]. Additionally, of the extracutaneous manifestations, only 3 case reports of splenic abscesses have been published [4], [5], [6]. Here we present a case of SS in an 18-year-old woman with rare sterile calcaneal and splenic abscesses seen on imaging.

## Case presentation

An 18-year-old woman initially presented for outpatient evaluation of hematochezia. Soon after, she developed bilateral foot and ankle pain, first on the right and subsequently on the left, with the pain progressing over the course of 2 months to the point where she could no longer bear weight.

She reported no history of recent upper respiratory infection, GI infection, or vaccination. She had no history of hematologic or visceral malignancy. She was previously healthy and took no prescribed medications. She reported no history of smoking, alcohol or drug use. Her urine pregnancy test was negative.

**Fig. 1:**
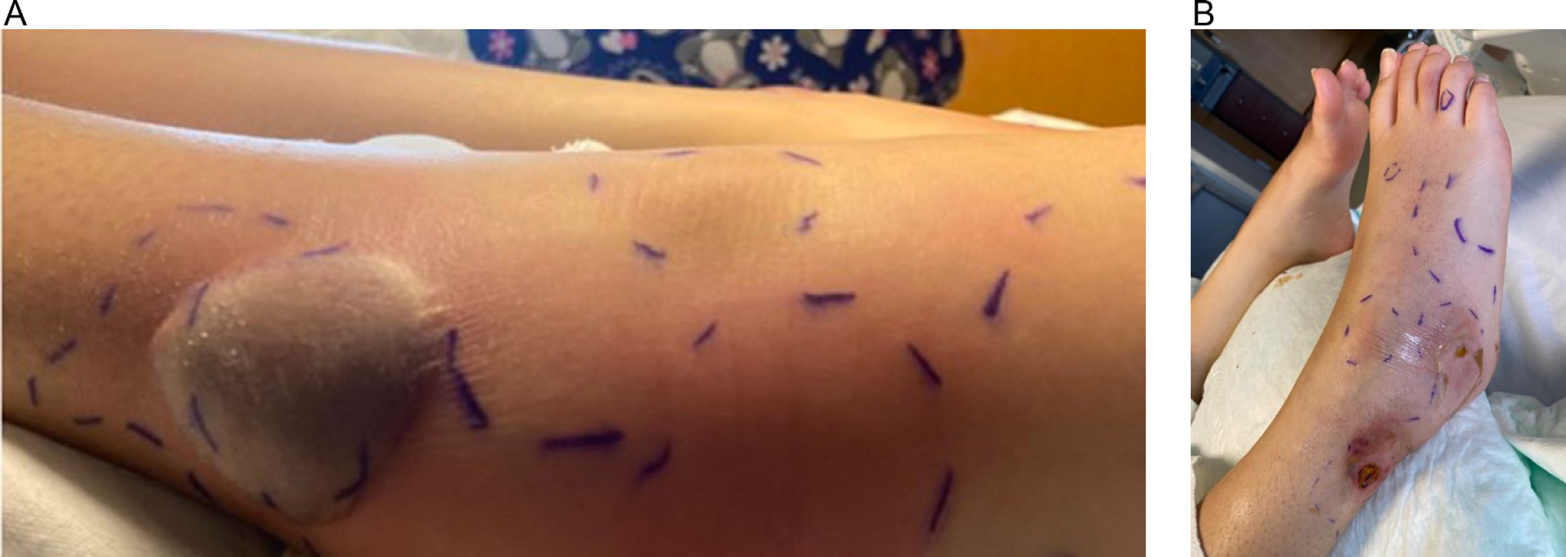
(A) Right lateral ankle unruptured fluid collection and surrounding erythema. (B) Right lateral ankle and foot swelling with spontaneous rupture of fluid collections.

**Fig. 2:**
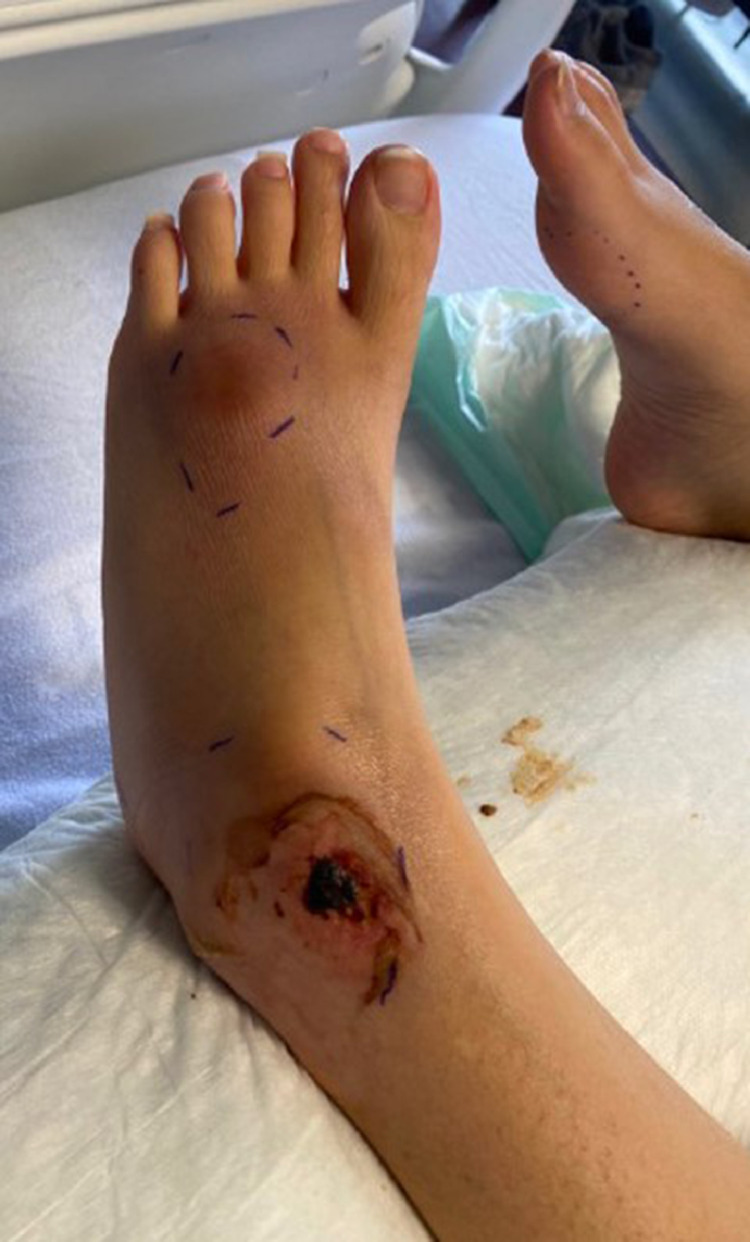
Left lateral ankle and foot swelling with spontaneous rupture of fluid collection over the left ankle.

**Fig. 3:**
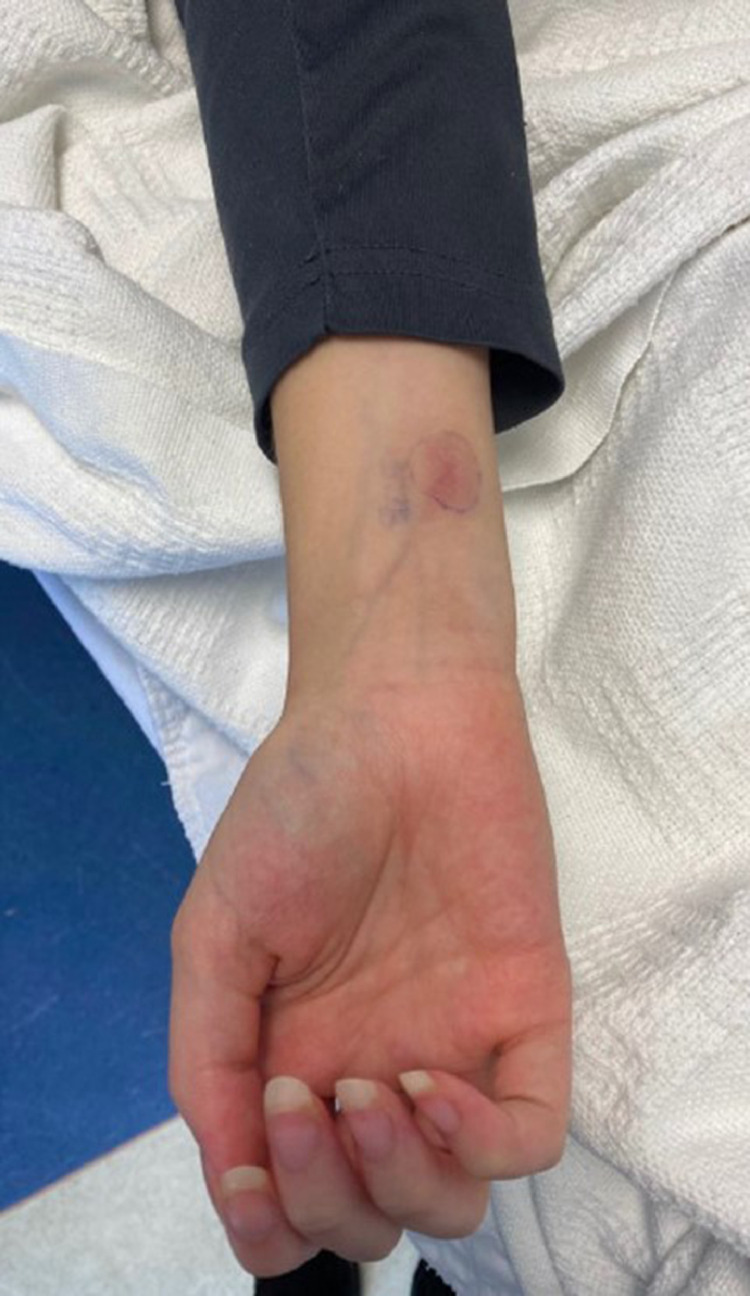
Right distal forearm erythematous plaque.

**Fig. 4:**
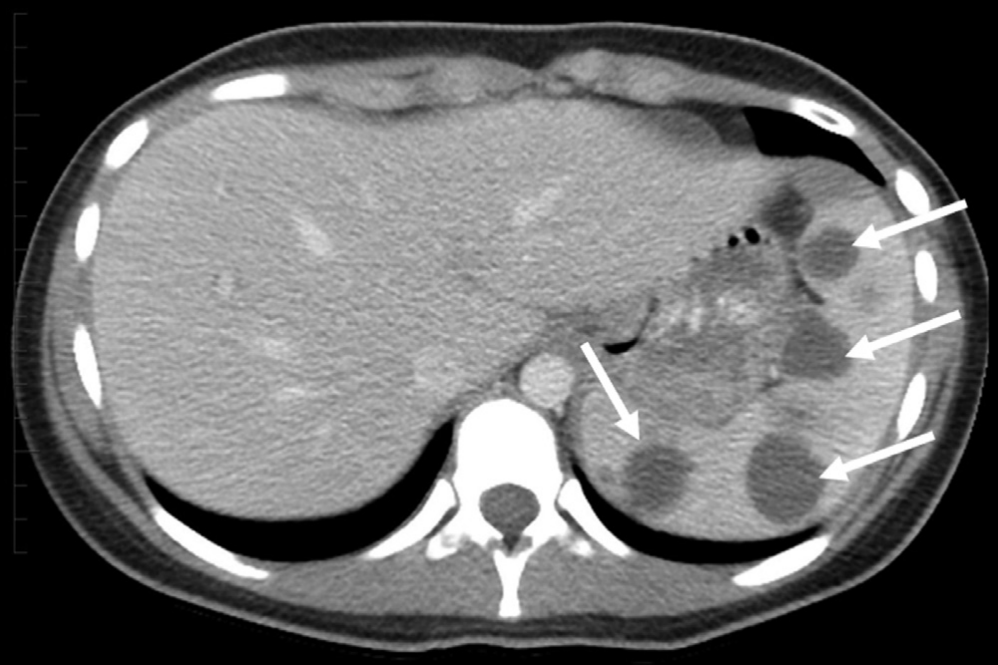
Axial CT with IV contrast demonstrating multiple round fluid collections in the spleen (arrows).

**Fig. 5:**
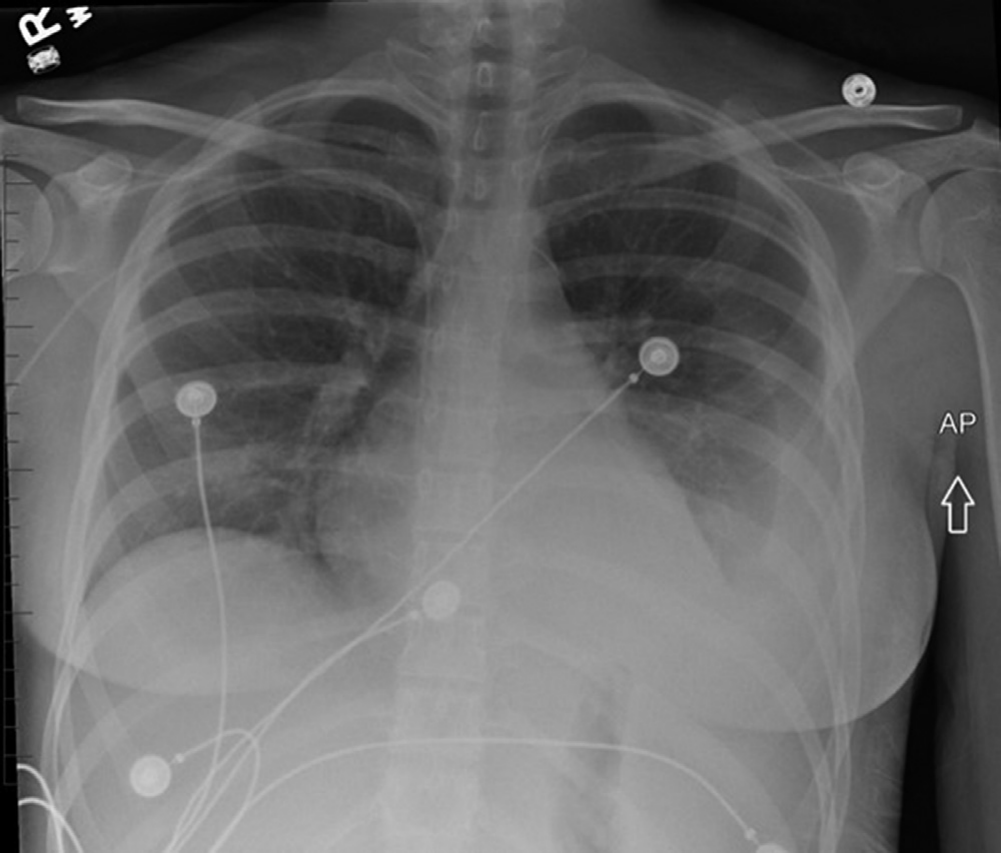
Subsequent chest radiograph demonstrated a moderate left pleural effusion. A right upper extremity PICC and multiple EKG leads are present.

The patient was admitted to the hospital due to worsening lower extremity pain, fevers (greater than 39°C), and tachycardia. Bilateral foot and ankle tenderness, warmth, and swelling were noted with several spontaneously draining fluid collections (Fig. 1, Fig. 2). An erythematous plaque was also seen in the right forearm (Fig. 3). Leukocytosis and elevated inflammatory markers were noted, including a WBC count of 19.4 K/uL with 81% neutrophils, CRP of 287 mg/L and ESR of 102 mm/hr. CT demonstrated splenic fluid collections and subsequent chest radiograph demonstrated a left pleural effusion (Fig. 4, Fig. 5). Transthoracic and transesophageal echocardiography were negative for any cardiac vegetations. Blood, pleural fluid, and splenic fluid cultures were negative, including aerobic, anaerobic, fungal, and AFB cultures. QuantiFERON-TB Gold, Parvovirus, Bartonella and Brucella antibody titers were also negative. Due to initial suspicion for infection, broad-spectrum antibiotics were started (vancomycin and piperacillin / tazobactam), but these were discontinued after the infectious workup was negative. p-ANCA was positive, which can be seen in several auto-immune conditions. Rheumatologic workup was otherwise negative, including ANA, RF, and complement levels.

**Fig. 6:**
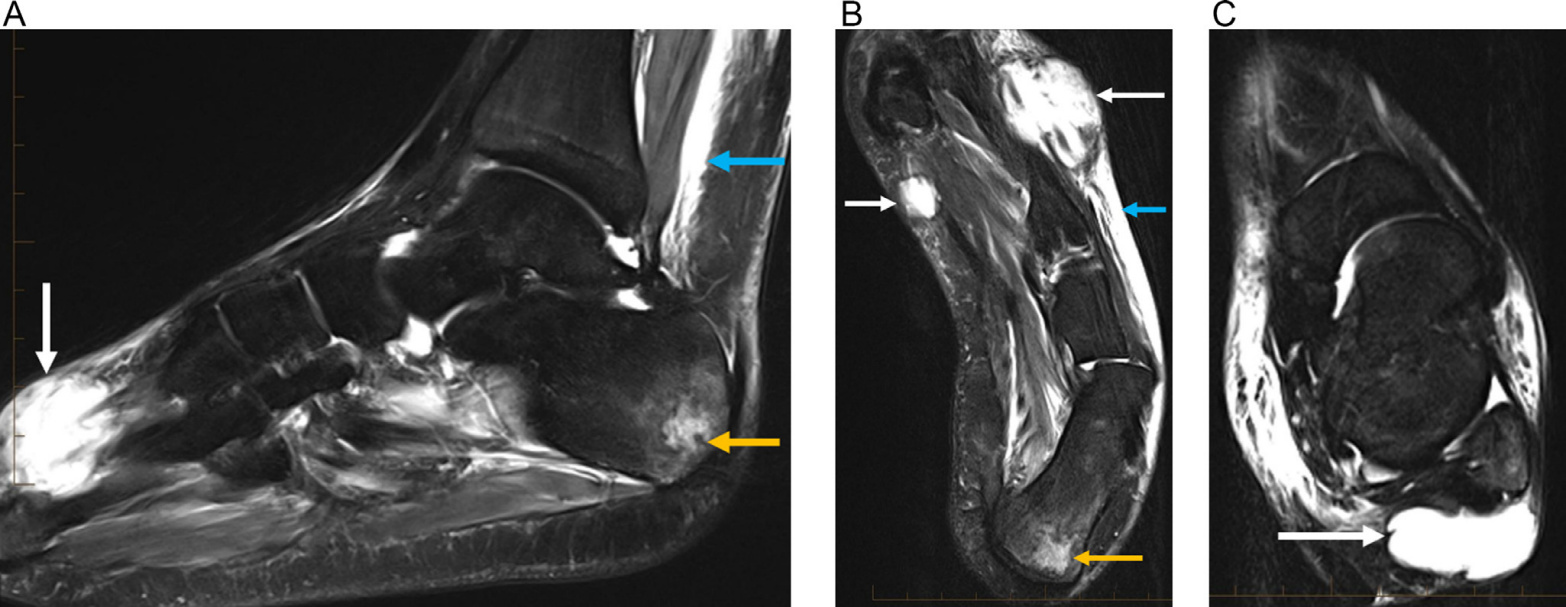
(A) Sagittal T2 fat sat image from baseline MRI of the left foot shows focal hyperintensity within the posterior calcaneus with surrounding ill-defined marrow edema pattern (orange arrow). This is not associated with the Achilles tendon or plantar fascia attachments. A focal fluid collection is seen in the dorsal soft tissues of the forefoot with traversing extensor tendons (white arrow). Edema is noted along Kager's fat pad (blue arrow). (B) Axial T2 fat sat image from baseline MRI of the left foot confirms focal marrow signal abnormality in the posterior calcaneus (orange arrow). Fluid collections are seen in the forefoot medially and laterally (white arrows). Subcutaneous edema is present laterally (blue arrow). (C) Axial T2 fat sat image from baseline MRI of the left foot demonstrates a focal fluid collection posterior to the lateral malleolus (arrow). Diffuse subcutaneous edema is noted (Color version of the figure is available online.)

**Fig. 7:**
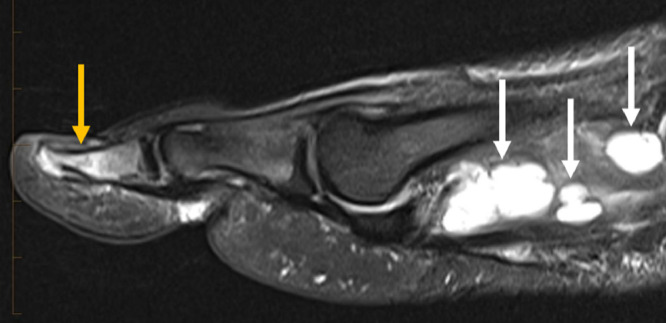
Sagittal T2 fat sat image from MRI of the right foot demonstrates edema-like marrow signal intensity in the hallux distal phalanx (orange arrow). Multiple fluid collections are noted in the forefoot (white arrows) (Color version of the figure is available online.)

MRI of the bilateral ankles and/or feet revealed multiple soft tissue fluid collections, focal fluid signal intensity within the left calcaneus with surrounding edema-like marrow signal intensity, and abnormal marrow signal in the right hallux distal phalanx tuft concerning for infection (Fig. 6, Fig. 7). However, the clinical picture and negative infectious workup prompted consideration of an inflammatory and/or autoimmune process. Punch biopsy of the right foot skin eruption revealed heavy dermal inflammation with abundant neutrophils, nuclear debris (leukocytoclasis), no vasculitic changes (fibrin deposition, necrosis or thrombosis of vessels), and no organisms, all of which suggested SS (Fig. 9).

**Fig. 8:**
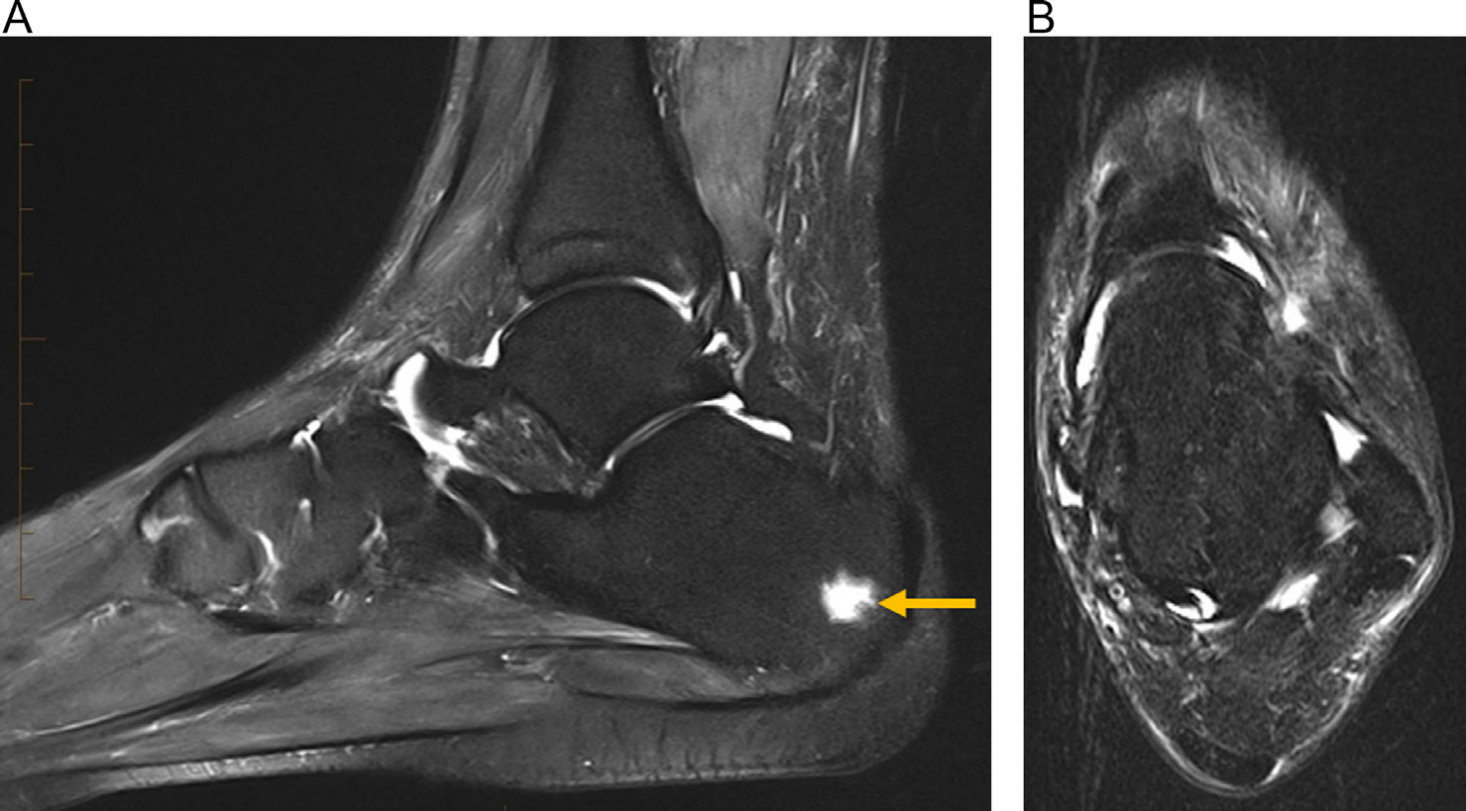
(A) Sagittal T2 fat sat image from follow up MRI of the left foot (3 months after the MRI shown in Fig. 6) demonstrates persistent focal marrow signal hyperintensity in the posterior calcaneus (arrow), although with interval resolution of surrounding marrow edema pattern and resolution of the previously noted soft tissue fluid collection. (B) Axial T2 fat sat image from follow up MRI of the left foot demonstrates complete resolution of the fluid collection that was previously seen posterior to the lateral malleolus.

The patient was started on systemic corticosteroids, initially IV prednisolone (50 mg daily for 3 days) and subsequently oral prednisone (40 mg daily). Her fever subsided and leukocytosis and CRP improved with steroids. The fluid collections in her feet markedly improved on clinical exam. Since SS may be associated with inflammatory bowel disease and since her symptoms started with hematochezia, she underwent a colonoscopy which revealed erythematous inflammation in the cecum. Biopsies of this area showed cryptitis, crypt abscess, and active and chronic colitis suggestive of Crohn's disease (Fig. 10). She underwent physical therapy, regained mobility, and was discharged home 3 weeks after admission. Subsequent MRI confirmed marked improvement in her left foot fluid collections (Fig. 8).

**Fig. 9:**
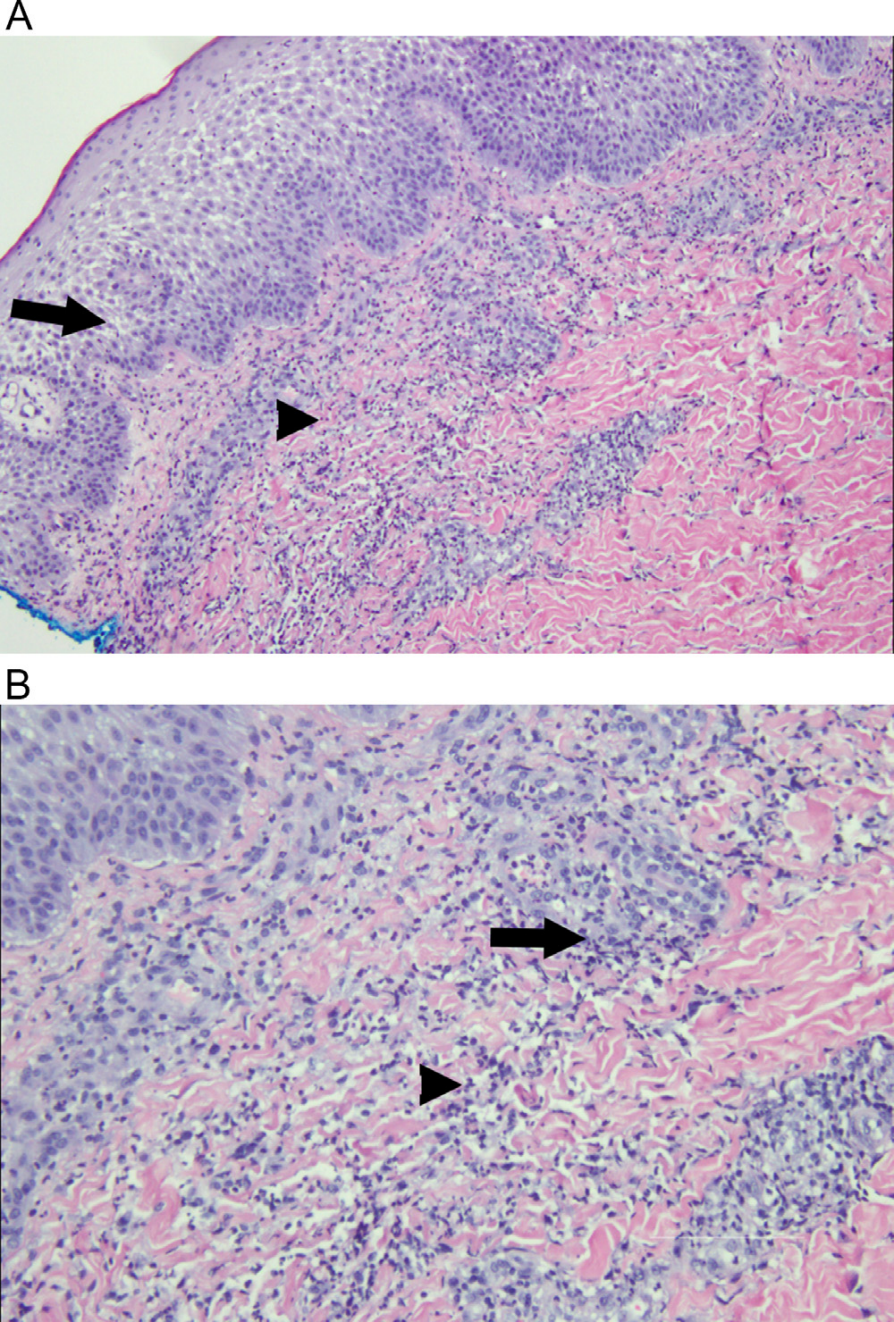
(A) Low power magnification of right foot punch biopsy showing epidermal edema (spongiosis) (arrow) and dense dermal perivascular and interstitial (arrowhead) inflammatory infiltrate with abundant neutrophils (H&E, 40x). (B) Higher power magnification of the dermal perivascular (arrow) and interstitial (arrowhead) inflammatory infiltrate with abundant neutrophils, nuclear debris, and no eosinophils (H&E, 200x).

**Fig 10:**
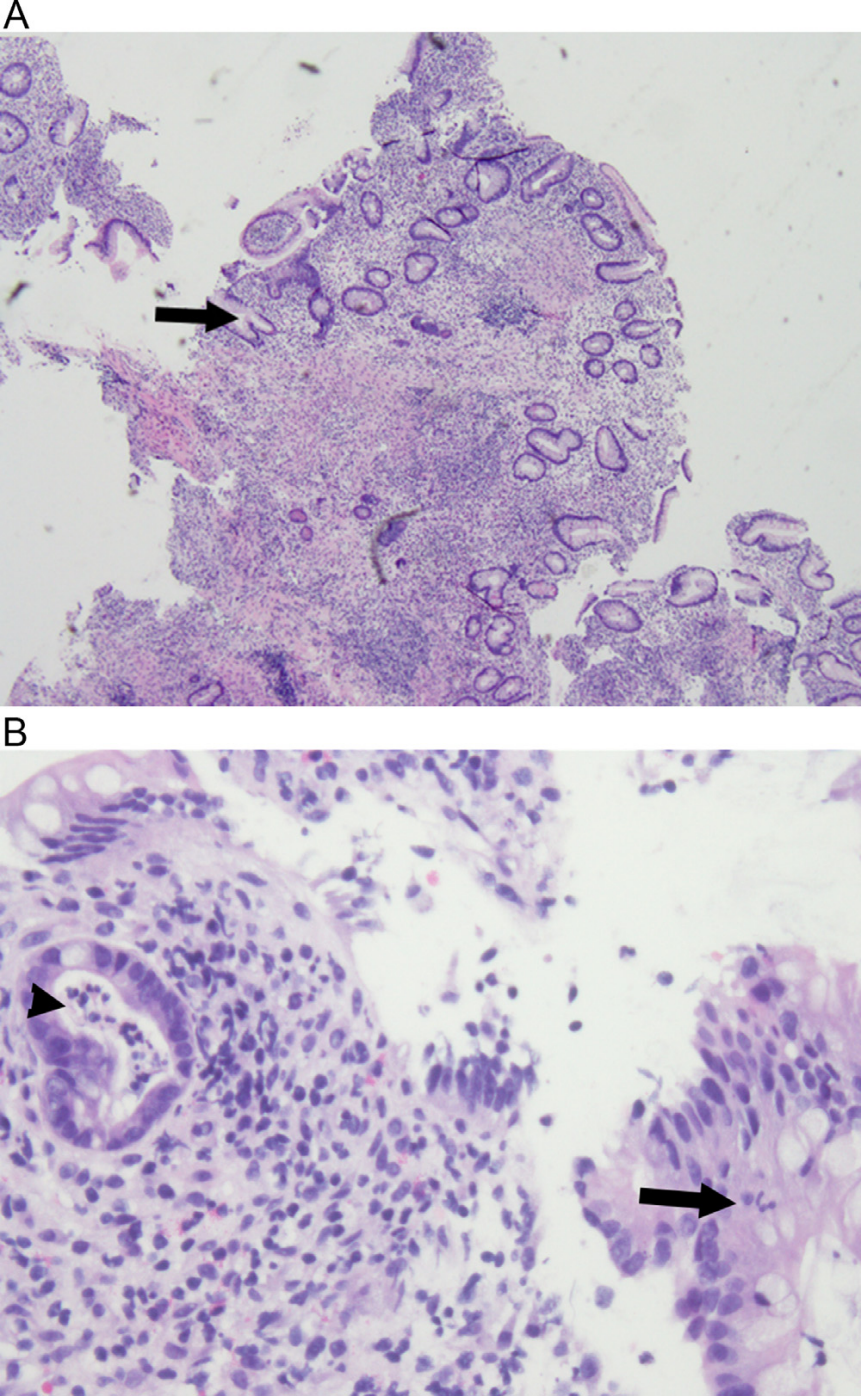
(A) Low power magnification of right colon biopsy showing chronic changes including shortened forked glands (arrow) with drop out and irregular distribution. There is a heavy chronic inflammatory infiltrate in the lamina propria (H&E, 40x). (B) High power magnification of right colon biopsy showing active inflammation including gland cryptitis (arrow) and crypt abscesses (arrowhead) (H&E, 400x).

A final diagnosis of hemorrhagic bullae secondary to SS in the setting of Crohn's colitis was made. The prednisone was ultimately tapered and discontinued, and the patient's symptoms continue to improve on scheduled adalimumab.

## Discussion

SS or acute febrile neutrophilic dermatosis is an uncommon disease characterized by an inflammatory and/or hypersensitivity reaction that most commonly manifests in the skin. The etiology is largely hypothesized to be autoimmune in nature. Its association with other systemic diseases and its constellation of clinical and histologic findings make it particularly challenging to diagnose [1,2,12].

Clinically, SS is characterized by the sudden onset of multiple tender, erythematous lesions (plaques, nodules or papules), fever, neutrophilic leukocytosis, and malaise. The dermatosis papules can range from erythematous to waxy and violaceous, and can then grow to form non-pruritic, painful and tender plaques on the face, neck, upper trunk, arms, hands, and feet [7,11,12,15,17]. Presenting similarly in both children and adults, SS typically has 2 age peaks: infancy and middle age. The syndrome can be classified into 3 categories based on presumed etiology: classical (idiopathic), malignancy-associated (usually acute myeloid leukemia), and drug-induced (usually granulocyte colony stimulating factor).

SS in children is more likely to be associated with preceding infection and tends to recur after tapering of corticosteroid treatment [7,11]. In adults, SS more commonly affects females, and an underlying malignancy has been described in 10-20% of cases. Classical Sweet syndrome is commonly preceded by an upper respiratory or GI infection, and may be associated with inflammatory bowel disease as seen in this patient [13]. The pathologic picture is that of a neutrophilic dermatosis in which abundant neutrophils are found in the reticular dermis with leukocytoclasis, edema and swelling of endothelial cells without vasculitis [1].

While arthritis, eye, and mucous membrane involvement are commonly reported in patients with SS, association with Crohn's disease is less common, and bone involvement and/or sterile bone abscess is very rare [8,12,14]. In the cases of SS associated arthritis, the literature has reported articular involvement to be asymmetric and migratory, more frequently involving the wrists and knees followed by ankles, elbows, and fingers [10]. We were unable to find any case reports mentioning true bone involvement other than malignancy. In one case study, chronic recurrent multifocal osteomyelitis (CRMO) and SS were postulated to be interconnected due to responsiveness to corticosteroid treatment and strong associations amongst related children [9]. However, CRMO is reported as an associated disease rather than an osseous manifestation of SS. Our patient's presentation of sterile calcaneal and splenic abscesses as manifestations of SS remains unique in the literature.

## Conclusion

Diagnosis of SS may be challenging and it is often a diagnosis of exclusion. In this case, negative infectious workup prompted consideration of an autoimmune process, and subsequent skin biopsy along with clinical history favored a diagnosis of SS. Improvement in clinical symptoms, laboratory markers, and imaging findings after treatment with corticosteroids further supported a diagnosis of SS. The patient met all major and minor criteria listed for classical SS [1,17]. From an imaging perspective, sterile abscesses should be considered in patients with a clinical diagnosis of SS and focal symptomatology. Imaging follow-up may be indicated to document treatment response, particularly for cases with osseous or visceral involvement.

## Patient consent

Formal consents are not required for the use of entirely anonymized images from which the individual cannot be identified - for example, x-rays, ultrasound images, pathology slides or laparoscopic images, provided that these do not contain any identifying marks, and are not accompanied by text that might identify the individual concerned.
